# Quantitative Assessment of Apically Extruded Debris During Retreatment Procedures Using Three Nickel-Titanium Rotary Systems: An In Vitro Comparative Study

**DOI:** 10.3390/dj12120384

**Published:** 2024-11-26

**Authors:** Luigi Generali, Federica Veneri, Francesco Cavani, Vittorio Checchi, Carlo Bertoldi, Angela Lucia Ingrosso, Giusy Rita Maria La Rosa, Eugenio Pedullà

**Affiliations:** 1 Department of Surgery, Medicine, Dentistry and Morphological Sciences with Transplant Surgery, Oncology and Regenerative Medicine Relevance (CHIMOMO), University of Modena and Reggio Emilia, 41124 Modena, Italy; luigi.generali@unimore.it (L.G.); federica.veneri@unimore.it (F.V.); carlo.bertoldi@unimore.it (C.B.); angelalucia.ingrosso@studio.unimore.it (A.L.I.); 2 Department of Biomedical, Metabolic and Neural Sciences, University of Modena and Reggio Emilia, 41124 Modena, Italy; francesco.cavani@unimore.it; 3 Department of General Surgery and Surgical-Medical Specialties, University of Catania, 95131 Catania, Italy; g_larosa92@live.it (G.R.M.L.R.); eugeniopedulla@unict.it (E.P.)

**Keywords:** apically extruded debris, HyFlex Remover, retreatment, ProTaper Retreatment, VDW.Rotate Retreatment

## Abstract

**Objectives**: Apical extrusion of debris can affect the success of endodontic treatments, and the specific performance of certain retreatment systems has not been studied yet. Therefore, the aim of this in vitro study was to quantitatively assess the amount of apically extruded debris produced during retreatment procedures using three rotary NiTi retreatment systems in mature non-resorbed straight roots. **Methods**: Thirty extracted permanent human teeth with single straight roots were selected. The root canals were prepared with the ProTaper Next system up to size 30 and obturated with gutta-percha and AH Plus sealer using the continuous wave of condensation technique. The samples were stored for 30 days and randomized by computer sequence into three retreatment groups (n = 10): (1) ProTaper Universal Retreatment; (2) HyFlex Remover; and (3) VDW.Rotate Retreatment. Apically extruded debris was collected in Eppendorf tubes and weighed with a microbalance (10^−5^ g) before and after retreatment procedure. As the data were not normally distributed, the Kruskal–Wallis test was applied for comparing data among groups, with an alpha level set at α = 0.05. Dunn’s test was considered for post-hoc analyses, if appropriate. **Results**: Hyflex Remover was associated with the highest amount of extruded debris (0.85 ± 0.82 mg), followed by VDW.Rotate Retreatment (0.78 ± 0.41 mg) and ProTaper Universal Retreatment (0.62 ± 0.28 mg). However, the differences were not statistically significant (*p* > 0.05). **Conclusions**: All the retreatment systems tested were associated with apical extrusion of debris in vitro, with no significant quantitative differences between them, suggesting that clinicians can choose a retreatment system with features appropriate to the specific clinical situation without risk of increasing the amount of apically extruded debris.

## 1. Introduction

A successful endodontic retreatment should be able to completely remove the existing intracanal obturation, in order to allow adequate cleaning, disinfection, and re-obturation of the root canal system, ideally without producing apically extruded debris [1,2,3,4].

The extrusion of infected tissue, dentin debris, irrigants, and root canal filling materials into the periapical tissues is regarded as a potential contributing factor to poor outcomes of endodontic treatments [1,5,6,7]. Apical debris extrusion has been associated with periapical inflammation, flare-ups, postoperative pain, delayed periapical healing, and long-term failures [6,7,8].

Seltzer and Naidorf [6] reported that a quiescent chronic inflammatory periapical lesion can evolve into a severe inflammatory reaction following the initiation of root canal treatment. The apical extrusion of debris during chemo-mechanical procedures, including microorganisms, exposes the periradicular tissues to a greater amount of irritants. This can lead to the disruption of the balance between microbial aggression and host defence typical of asymptomatic chronic periradicular lesions, resulting in an acute inflammatory response within the periradicular tissues [6,7]. Although the presence of virulent microorganisms is an important causal factor in the occurrence of flare-ups, it is also recognized that pulpal and dentin debris, whether contaminated or not, have the potential to initiate an inflammatory response [9]. In a study by Seltzer et al. [10], uncontaminated dentin debris was extruded past the apex during overinstrumentation, and caused painful distension of the collagen fibers of the periapical periodontal ligament. Furthermore, based on in vivo animal experiments, biocompatible obturation materials, such as gutta-percha, are considered well tolerated by tissues [11]. However, clinical observation has associated extruded gutta-percha with delayed healing of the periapical region. By implanting sterile gutta-percha into the subcutaneous tissue of guinea pigs, it has been observed that larger sized particles with a smooth surface tend to be well encapsulated by collagen and the surrounding tissue remains inflammation-free. In contrast, small gutta-percha particles induce an intense local tissue response with macrophages and giant cells [11,12]. Although clinicians have little or no control surrounding the qualitative composition of the debris, the amount of extruded debris can be ideally reduced by choosing appropriate preparation and irrigation techniques. For example, rotary and reciprocating instruments used in a crown-down technique, and the use of side-vented needles for irrigation, seem to cause less debris extrusion than hand instruments [9,13].

Certain retreatment systems have been available for a considerable period of time and have been subjected to a larger number of studies, thereby providing a basis for comparison and reference. For example, the ProTaper Universal Retreatment (PTU-R, Dentsply Sirona, Ballaigues, Switzerland) system is a well-established multiple-file system that includes three files (D1, D2, and D3) with convex and triangular cross-sections. The D1 (size 30, 0.09 taper, 16 mm in length) has an active working tip and is used in the cervical third of the root canal. The D2 (size 25, 0.08 taper, 18 mm in length, non-active tip) is used in the middle part of the canal system, and the D3 (size 20, 0.07 taper, 22 mm in length, non-active tip) is designed to remove the filling material along the entire length of the canal [1,14].

Continuous innovations in endodontic alloys, manufacturing processes and instrument designs have led to the introduction of new file systems with distinct features that are thought to potentially influence apical extrusion of debris, such as the number of instruments, specific sequence of approach to the apex, kinematics, and file design [13,15].

Among the most recent file systems introduced for retreatment procedures, two single-file systems have become available: Hyflex Remover (HF-R, Coltene/Whaledent AG, Altstätten, Switzerland) and VDW.Rotate Retreatment (VDW.R-R, VDW, Munich, Germany). Hyflex Remover consists of one heat-treated file (size 30, 0.07 taper), available in two lengths (19 and 23 mm), designed with a triple helix section and a non-active tip which ensures the respect of the canal anatomy and reduces the occurrence of procedural errors. The VDW.Rotate Retreatment file is available in 25 size and 0.05 taper and is 21 mm in length, made from a traditional NiTi alloy with an active tip and a modified S cross-section designed for intracanal obturation removal. Previous studies have evaluated the occurrence of apical extrusion with ProTaper Universal Retreatment [16,17] and found slight or no significant differences with a single-file reciprocating system and other rotary systems, although different sequences were tested.

Two studies have recently investigated the amount of apically extruded debris in teeth with simulated apical root resorption using more recent HyFlex Remover and VDW.Rotate Retreatment instruments [18,19]. Çağlar et al. [18] found no significant differences between VDW.R-R and ProTaper Universal Retreatment (PTU-R), while Gayatri et al. [19] reported a significant lower amount of apically extruded debris for PTU-R compared to HF-R [1,14].

However, no data are currently available regarding apically extruded debris using the aforementioned retreatment systems in non-resorbed roots.

Therefore, the purpose of this in vitro study was to quantitatively assess the amount of apically extruded debris produced by using three different NiTi retreatment systems (ProTaper Universal Retreatment, HyFlex Remover, and VDW.Rotate Retreatment) in mature non-resorbed straight roots. Given the common consensus that no instrumentation system is capable of preventing debris extrusion, the null hypothesis chosen stated that there would be no significant differences in the amount of debris extruded among the three NiTi systems.

## 2. Materials and Methods

### 2.1. Sample Selection

Sample size was estimated according to previous studies [15,20] and the minimum sample size resulted in 10 teeth per group for a test power of 0.80 (G*Power 3.1.9.2 software, Heinrich-Heine-Universität Düsseldorf, Düsseldorf, Germany), with α = 0.05.

Thirty single-root, permanent human teeth were selected from an anonymized biobank of teeth extracted for periodontal reasons. The exclusion criteria were: open apices, initial apical diameter greater than a size #25 K-file [21], resorptive defects or calcifications, caries, and previous root filling fractures or cracks verified using a stereomicroscope (OPMI Pico; Carl Zeiss Meditec Inc., Jena, Germany) (10× magnification). Selecting roots with an apical diameter of up to #25 k-file reduces the risk of undetected apical resorption or apex immaturity, helps standardize the baseline risk for apical debris extrusion, and better simulates real-life clinical situations [19].

Schneider’s method [22] was used to measure the degree of canal curvature using digital radiographs acquired in buccolingual and mesiodistal directions. Only roots with a degree of curvature less than 10° were selected.

All the soft and hard tissue residues on the root surfaces were removed by scraping the surface with periodontal instruments. All teeth were disinfected in a 5.25% NaOCl bath and subsequently stored in a 0.1% thymol solution at 5 °C for 30 days [23].

### 2.2. Root Canal Preparation and Obturation

Preparation, obturation, and obturation removal procedures were performed by the same experienced endodontist using a dental operating microscope (OPMI Pico) at 10× magnification.

All the specimens were subjected to the same primary endodontic treatment protocol, ensuring that no variations in the preparation procedures occurred, to avoid possible biases in the results. In detail, for all the specimens, the crowns were sectioned using a diamond disk to standardize the samples with 13 ± 1 mm canal length. A #10 K-file was inserted until it protruded 1 mm through the major foramen to confirm patency and to standardize the apical diameter for all teeth. Working length (WL) was measured by inserting a 21 mm #10 K-file (Dentsply Maillefer, Ballaigues, Switzerland) until its tip appeared at the apical foramen under ×10 magnification. The mechanic glide path was obtained with ProGlider (size 16, 0.02 taper) (Dentsply Sirona, Ballaigues, Switzerland). Afterwards, root canals were instrumented with X1 (size 17, ~0.04 taper), X2 (size 25, ~0.06 taper), and X3 (size 30, ~0.07 taper) 25 mm-long Pro-Taper Next NiTi instruments (Dentsply Sirona), following manufacturer recommendations. During this procedure, an X-Smart Plus (Dentsply Sirona) endodontic motor was used. During instrumentation, 5 mL 2.5% sodium hypochlorite (NaOCl) and for final irrigation 5 mL 17% ethylenediaminetetraacetate (EDTA) and 5 mL 2.5% NaOCl solutions were used. All irrigating solutions were delivered with a 30 G side-vented irrigation needle (Max-i-Probe; Dentsply Rinn, Elgin, IL, USA) placed 1 mm short of the working length (WL).

The root canals were dried using paper points and obturated with a ProTaper Next Conform Fit X3 gutta-percha cone (Denstply Maillefer, Baillague, Switzerland) and AH Plus sealer (Dentsply De Trey, Konstanz, Germany) through the continuous wave of condensation technique [24]. After placing the single cone, the condensation technique was performed with a heated plugger (Kerr/SybronEndo, Orange, CA, USA) to 3 mm from the WL of each tooth, and the remaining coronal root canal space was filled through injected gutta-percha from a Backfill device (Elements Free Obturation System, Kerr/SybronEndo, Orange, CA, USA). Access cavities were finally filled with Cavit (3M ESPE, Seefeld, Germany). Digital radiographs were acquired in the buccolingual and mesiodistal directions to verify appropriate root canal obturation. Radiographically, none of the samples showed voids within the obturation. All teeth were stored at 37 °C in 100% humidity for 25 days to allow the sealer to set [4].

### 2.3. Retreatment Procedure and Debris Collection

The experimental design described by Myers & Montgomery was used to collect the apically extruded debris [25]. A digital microbalance (AE240, Mettler-Toledo; Columbus, OH, USA) with an accuracy of 10^−5^ g was used to weigh the Eppendorf tubes after removing the stoppers. For each Eppendorf tube, six measurements were taken, and the average was calculated in order to achieve a greater reliability and accuracy of measurements [25,26,27]. The manipulation of the Eppendorf tubes was conducted with extreme care, utilising only clean gloves or tweezers to prevent contamination and ensure consistency across measurements. A round hole was made in the stoppers removed from the Eppendorf tubes. The teeth were positioned into the stopper up to the cement–enamel junction and fixed with cyanoacrylate (Pattex Super Glue; Türk Henkel, Inc., Istanbul, Turkey) to avoid the leakage of irrigants. In order to balance the internal and external air pressures, a 25 G needle was inserted in the stopper.

**Figure 1:**
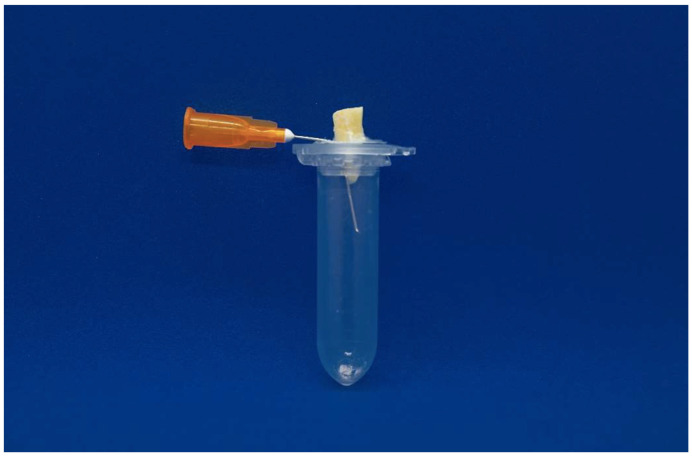
Eppendorf tube with root sample and 25 G needle fixed to vial stopper to balance internal and external pressures.

The stoppers, including the teeth and needles, were then secured to Eppendorf tubes, and the tubes were inserted into vials. The vials were covered with a rubber dam sheet to prevent the operator from observing the root apex during the retreatment procedure *(*Figure 1) [27].

The specimens were numbered and randomly allocated to three groups (n = 10) using a web-based algorithm generating random sequences (www.random.org). Due to technical reasons, the operator performing the endodontic procedures was not blinded to the system used. The files were operated in an endodontic motor (X-Smart Plus, Dentsply Sirona) as follows, according to manufacturer recommendations for each system: (1) PTU-R: the D1 file was employed for retreatment of the coronal third, the D2 file for the middle third, and the D3 file was inserted at working length. All three instruments were activated at 500 rpm and 2.5 Ncm. Apical preparation was carried out with Pro-Taper Next X2, X3, and X4 (size 40, 0.06 taper), at 300 rpm and 2 Ncm; (2) HF-R: the single file HyFlex Remover was used for the removal of root canal obturation up to 3 mm from the apex, at 400 rpm and 2.5 Ncm. The final apical preparation was obtained with Hyflex CM (Coltene) files #30 (size 30, 0.04 taper) and #40 (size 40, 0.04 taper) at 500 rpm and 2.5 Ncm; (3) VDW.R-R: the VDW Rotate Retreatment instrument was used at WL, at 400 rpm and 3.5 Ncm, to gradually remove the root canal obturation with circumferential movements. The final apical preparation was performed with VDW.Rotate files (VDW) #30 (size 30, 0.04 taper) and #40 (size 40, 0.04 taper) at 300 rpm, and 2 and 2.3 Ncm, respectively.

The temporary filling was removed using a round bur. No solvent was employed during the retreatment procedure. When the working length could not be reached by rotary instruments, a stainless-steel size 15 file was used to negotiate the root canal, as in similar studies, which provided a good compromise between file efficacy and reduced influence on apical debris production [28,29,30].

**Figure 2:**
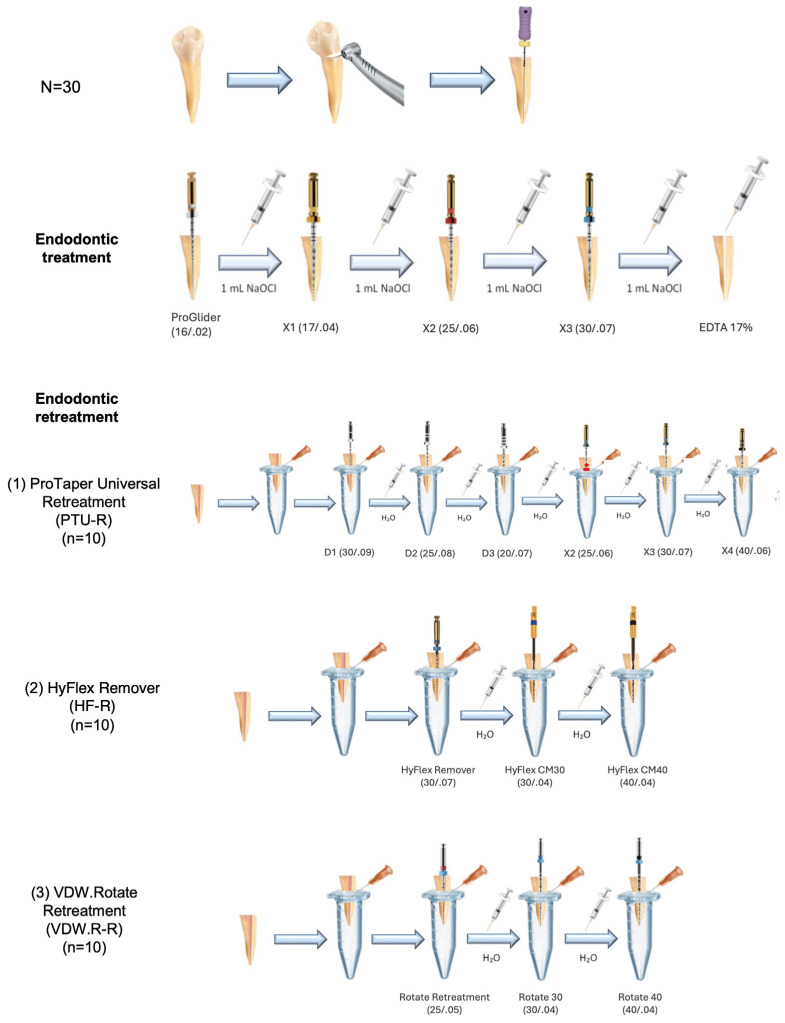
Schematic of the experimental procedures, including primary endodontic treatment and retreatment.

A total of 10 mL distilled water was used for each canal as irrigant during instrumentation, using a 30 G side-vented irrigation needle, inserted 2 mm short of working length. All the instrumentation and irrigation procedures were standardized across the groups to ensure consistency. A schematic of the procedures is provided in Figure 2. The potential occurrence of perforations, instrument fractures, and blockages during the treatment and retreatment procedures was verified by visual inspection under a dental operating microscope (OPMI Pico) at 10× magnification and eventually recorded as procedural incidents. A complete removal of the root canal filling material was considered achieved when the working length was reached and no residual obturation material was observed on the instrument flutes or in the irrigation solution when examined with a magnifying loupe (4×) [4]. The Eppendorf tubes were then removed from the vials and the apical portion was rinsed with 1 mL of distilled water to collect the extruded debris that had adhered to the apex [27].

Eppendorf tubes were kept in an incubator at 37 °C for three weeks to allow evaporation of the distilled water and then weighed again on a digital microbalance to an accuracy of 10^−5^. As previously, the measurements were performed six times for each Eppendorf tube, and averaged. The net weight of the dry debris was obtained by subtracting the weight of the empty Eppendorf tube from the final weight [27]. Measurements and calculation of apically extruded debris were performed by an operator blinded to the retreatment group allocation of the specimens.

### 2.4. Statistical Analysis

Statistical analyses were conducted using Stata 16.1 (StataCorp LLC, College Station, TX, USA) software. Normality was assessed using the Shapiro–Wilk test. As the data were not normally distributed, the non-parametric Kruskal–Wallis test was applied to compare the amount of debris extrusion among retreatment groups. Dunn’s test was considered for post-hoc analyses, if significant differences were found. The statistical significance level was set at an alpha level of 0.05.

## 3. Results

The mean values and standard deviation of the apically extruded debris measured in milligrams (mg) of each experimental group are displayed in Table 1, along with the median values and range. All the instruments were associated with extrusion of the debris from the apical foramen. No significant difference was found regarding the amount of extruded debris among the three groups (*p* > 0.05). Therefore, post hoc analyses were not performed. No procedural errors (e.g., perforations, instruments fractures, or blockage) occurred, neither during the treatment nor the retreatment procedures.

## 4. Discussion

A suitable instrument for endodontic retreatment should allow for the complete removal of the root canal filling in a short intervention time, without causing adverse effects, instrument fractures, alteration of the root canal anatomy, or the apical extrusion of debris.

Apical extrusion of debris has clinical and biological importance, as it can be associated to post-operative complications such as inflammatory foreign-body reactions, pain relapses, and unresolved infectious environments, thus leading to possible failure of endodontic therapy [9,31].

According to the literature to date, no system can prevent apical debris extrusion [13,32,33,34], but some characteristics of NiTi files, such as kinematics, cross-sectional geometry, tip size and taper, and thermal treatment, have been considered as influencing factors on the amount of debris extruded [34,35].

For example, some retreatment techniques have been associated with a higher amount of apical debris formation. While most studies have found no significant differences between rotary NiTi systems and hand instruments, in terms of effectiveness in removing the existing root canal filling, many studies have observed significantly greater debris extrusion using H-file manual instruments compared to rotary and reciprocating systems [26,36]. This can be explained by the fact that H-files are used with a manual “push-pull” instrumentation technique, which might increase the risk of debris ejection through the apical foramen, whereas rotary NiTi instruments combine a rotary movement with a pressure-free action, and they are able to collect debris, conveying it in the coronal direction [31,33].

In the present study we evaluated the amount of extruded debris produced by three NiTi systems. The tested instruments have different recommended operative parameters, kinematics, tip size, and taper, and only some of them are thermally treated. Despite these variations, all retreatment systems caused apical extrusion of debris, confirming the general consensus in the literature. The Hyflex group was associated with a slightly higher amount of debris extrusion, followed by the Rotate and, lastly, the ProTaper group, although no significant difference (*p* > 0.05) was found amongst the three NiTi systems, so the null hypothesis was accepted.

A first observation is that these findings corroborate the fact that the number of instruments does not seem to affect the amount of apically extruded debris, as the tested single-instrument systems do not prevent debris extrusion, similarly to what has been reported for all other current instrumentation techniques [9,34,35]. This may support the choice of single-instrument systems in selected clinical situations, whose advantage should be to achieve a simplified protocol, leading to a reduction in intervention time [35,36]. Nevertheless, some studies have reported that the time required to perform endodontic retreatment with single-file systems may be similar or even longer than using multiple-file systems, especially in curved roots [37,38].

Conversely, other authors have instead reported a greater amount of apically extruded debris with multi-instrument systems compared to single-instrument systems, but the different rotating and reciprocating kinematics of the systems tested, as well as the number of files, might have played a concurrent role in determining the debris production [39,40].

Such discrepancies in results could be explained by operator-dependent factors and methods used to measure the intervention time during retreatment procedures.

The final apical diameter and taper achieved was also evaluated as a possible factor influencing the amount of apically extruded debris. The root canal preparation should exceed the size of the primary shape, and an enlargement of apical preparation is recommended in order to completely remove the obturation material, delineate a cleaner root canal preparation, especially in the apical third [41], and create adequate space for effective irrigation [27,42]. This is essential to control infection and obtain long-term success [43].

In the present study, an additional apical preparation was performed after removing the root canal filling to ensure that the final apical diameter was homogeneous in all experimental groups. In this study, the final apical diameter obtained with the three NiTi systems was 0.40 mm. Even if the taper of the last instruments was different (0.06 and 0.04 respectively), this parameter did not seem to influence the amount of debris extruded.

Concerning the flexibility of the analyzed systems, differently from the traditional heat-treated M-wire alloy of PTU-R and VDW.R-R, Hyflex Remover features a heat-treated controlled-memory wire (CM-Wire), which provides it with greater flexibility, allowing the preservation of the canal curvature as well as increasing efficiency and safety during instrumentation [44].

Some authors have reported a significantly lower debris extrusion using HF-R compared to PTU-R in teeth with curved roots [45]. On the other hand, in the study by Capar et al. [46] on straight or slightly curved roots, a higher amount of debris using HF-R than PTU-R was reported [46]. In a recent study comparing resorbed and non-resorbed roots, HF-R was found to produce a significantly greater amount of debris than PTU-R in both groups [19]. Similarly, our study on straight roots reported a slightly higher extrusion of debris using HF-R. Although the difference was not statistically significant, this finding may be explained by the non-cutting tip of the HF-R system, which may force debris toward the apex [44]. Additionally, rather high standard deviations and a wide range of values were recorded, especially in HF-R group, that may affect the interpretation of the mean values, indicating more variability and less certainty about the representativeness of the mean for the group.

The experimental setting used in this study, firstly described by Myers and Montgomery, has been widely adopted for investigations on apical extrusion of debris during endodontic procedures [25]. Nevertheless, the discrepancies between the weight of debris and the results reported by different authors using the same experimental setting are a clear indication of the complexity in the standardization of these studies.

In this regard, it is also worth mentioning that many studies exclude teeth with moderate curvatures to maintain uniformity and reduce biomechanical variability in shaping and cleaning the apical third. Similarly, in the present study, we aimed at assessing debris extrusion in straight roots (with < 10° curvature as established by Schneider’s method and as adopted by other authors) [22,46]. Such restrictions on canal curvature and apical diameter aim to simulate typical clinical scenarios while maintaining experimental consistency. In fact, excessive canal curvature can introduce variability in debris extrusion due to altered flow dynamics and increased difficulty in maintaining a uniform working length and instrumentation path, as well as increase the risk of procedural errors such as ledging or apical transportation, which may affect the results [22,46].

Overall, these findings have potential implications for clinical decision making, suggesting that clinicians can select a retreatment system that is tailored to the specific clinical scenario, including the clinician’s experience and training with specific instruments, without concern that it will significantly influence the amount of apically extruded debris.

However, these results must be considered with caution, particularly in light of a number of limitations inherent in the in vitro study design. First, a critical aspect of the present methodology is the impossibility of preventing the possible contamination of the collection devices during all the experimental phases, that may induce variations in the measurements [39]. Solda et al. [20] in their recent study tried to overcome this limitation by using a collection system with paper filters. However, this system only allowed the collection of solid debris, precluding the possible measurement of extruded irrigants. In the present study, we adopted a careful manipulation of the samples to limit this issue, while using a method that allows for a comprehensive evaluation of extruded debris.

The phase of samples drying is also a weak point of the experimental methodology, as the time and temperature parameters of storage are extremely variable in the literature and the drying is highly dependent on the humidity and heat of the environment in which the samples are stored. It is also not possible to guarantee an identical amount of drying and evaporation for all samples [9]. Another limitation in reproducing a real clinical situation in this experimental model is the absence of irrigants that are commonly used during retreatment procedures, such as NaOCl and/or EDTA. However, similarly to other authors, double-distilled water was used as the irrigant in the present study because, unlike water, NaOCl and EDTA can form into crystals during evaporation. The crystal precipitates cannot be separated from debris and may affect the results of the study, leading to overestimation of the amount of extruded debris [47]. Such heterogeneity in experimental procedures could be overcome by the possible future identification of an ISO standard for this type of study.

A major limitation of this study is the absence of materials around the teeth simulating the periapical tissue resistance to the extrusion of debris. The use of floral foam and agar gel has been reported in the literature, but there are adverse effects and difficulties in using the correct thickness to properly simulate periapical resistance [27]. In addition, some authors have pointed out that the condition of the pulp, whether vital or necrotic, and the possible presence of periapical lesions may also influence the amount of extruded debris and irrigant [26,48].

Future research should be conducted on the complementary evaluation of the efficacy in removing the filling material using modern technologies, such as micro computed tomography. This could provide additional insight into the overall performance of these retreatment systems and further guide the clinical choice of specific retreatment systems, also in consideration of the different existing filling material to be removed [49,50,51]. Finally, a purely quantitative assessment of apical extrusion may be of limited clinical relevance. The assessment of the amount and of the potential clinical impact of debris deriving from different obturation materials and techniques also have important clinical relevance, as well as the characteristics of the pulp tissue [51]. In fact, flare-ups were found to be significantly more frequent in cases with necrotic (presumably infected) pulp than in cases with vital (presumably uninfected) pulp [5,6]. Additionally, the amount, type, and virulence of bacteria bound to the debris may also modulate the periapical response [7]. Extruded bacteria are the main pathological component of the debris, especially when E. faecalis is present, which is often associated with endodontic treatment failures [52]. Thereby, further studies should include a qualitative analysis of the content of the extruded material, investigating obturation materials, pulpal tissue, and bacterial components [53].

To deepen our understanding of the performance of these retreatment systems, further research is essential, specifically studies focused on the effectiveness of these systems in thoroughly removing existing obturation material, alongside detailed qualitative analyses of the extruded debris.

## 5. Conclusions

All of the NiTi systems analyzed in this study equally produced apical extrusion of debris during retreatment procedures. These findings indicated that the distinct designs and characteristics of the files did not significantly impact the amount of debris extruded. Within the limitations of this in vitro study, these results suggest that clinicians can choose a retreatment system with features appropriate to the specific clinical situation without risk of increasing the amount of apically extruded debris. Further studies evaluating the effectiveness in the removal of the existing obturating material and additional qualitative analyses of the extruded debris are encouraged to gain a more detailed understanding of the performance of the retreatment systems. Such research would provide critical insights into optimizing endodontic procedural standards and improving retreatment outcomes.

## Figures and Tables

**Table 1:** Mean values and standard deviations (SD) of apically extruded debris in milligrams (mg).

Retreatment Group	Extruded Debris (mg)		
	Mean ± SD	Median	Range
1. PTU-R	0.62 ± 0.28 ^a^	0.55	0.20–1.01
2. HF-R	0.85 ± 0.82 ^a^	0.63	0.16–2.93
3. VDW.R-R	0.78 ± 0.41 ^a^	0.74	0.15–1.46

SD: standard deviation; different superscript letters indicate a statistical significance within the column (*p* < 0.05).

## Data Availability

Any additional data supporting these findings are available from the corresponding author upon reasonable request.
